# Biosensing of Haemorheological Properties Using Microblood Flow Manipulation and Quantification

**DOI:** 10.3390/s23010408

**Published:** 2022-12-30

**Authors:** Yang Jun Kang

**Affiliations:** Department of Mechanical Engineering, Chosun University, 309 Pilmun-daero, Dong-gu, Gwangju 61452, Republic of Korea; yjkang2011@chosun.ac.kr; Tel.: +82-62-230-7052; Fax: +82-62-230-7055

**Keywords:** haemorheological property, blood viscosity, blood viscoelasticity, RBC aggregation, blood flow quantification

## Abstract

The biomechanical properties of blood have been used to detect haematological diseases and disorders. The simultaneous measurement of multiple haemorheological properties has been considered an important aspect for separating the individual contributions of red blood cells (RBCs) and plasma. In this study, three haemorheological properties (viscosity, time constant, and RBC aggregation) were obtained by analysing blood flow, which was set to a square-wave profile (steady and transient flow). Based on a simplified differential equation derived using a discrete circuit model, the time constant for viscoelasticity was obtained by solving the governing equation rather than using the curve-fitting technique. The time constant (*λ*) varies linearly with respect to the interface in the coflowing channel (*β*). Two parameters (i.e., average value: <λ>, linear slope: $\frac{d\lambda }{d\beta }$) were newly suggested to effectively represent linearly varying time constant. <λ> exhibited more consistent results than $\frac{d\lambda }{d\beta }$. To detect variations in the haematocrit in blood, we observed that the blood viscosity (i.e., steady flow) is better than the time constant (i.e., transient flow). The blood viscosity and time constant exhibited significant differences for the hardened RBCs. The present method was then successfully employed to detect continuously varying haematocrit resulting from RBC sedimentation in a driving syringe. The present method can consistently detect variations in blood in terms of the three haemorheological properties.

## 1. Introduction

Blood consists of various types of cells (red blood cells [RBCs], white blood cells [WBCs], and platelets) and liquid plasma [1]. As the number of RBCs is significantly larger than that of other cells (i.e., RBCs: 4.3–5.7 million per µL, WBC: 4000–11,000 per µL, and platelet: 150,000–400,000 per µL), dynamic blood flow is determined predominantly by the volume and biomechanical characteristics of RBCs. Haematocrit (Hct) is defined as the RBC volume in relation to the overall blood volume. It is considered one of the vital factors that influence the change in haemorheological properties. Owing to their extremely high deformability, RBCs can easily pass through narrow capillary channels for effective mass transport (i.e., gas exchange, nutrients, and wastes) between capillary channels and peripheral tissues. Liquid plasma contributes to a varying viscosity and aggregation. Previous studies have reported that coronary heart diseases significantly alter haemorheological properties [2,3]. Various haemorheological properties (i.e., viscosity, deformability, and aggregation) have been measured simultaneously to detect variations in RBCs and plasma [4,5,6,7,8]. Blood viscosity is quantified by measuring pressure-related parameters (i.e., interface relocation [9,10] or fluidic resistance variation [11,12,13]) under constant shearing blood flow. By supplying a single RBC into a microfluidic channel, RBC deformability can be assessed by monitoring transient characteristics [14,15], impedance variation [11], RBC velocity [16,17], RBC clogging [18], and cortical tension [19]. When blood flows in the microfluidic channel (i.e., channel dimension: 10–100 µm) with a syringe pump, the shear rate is estimated to be sufficiently high (i.e., $\dot{\gamma \;}$> 10^3^ s^−1^). Blood behaves as a Newtonian fluid. RBCs are aligned and deformed in a single flow direction. At higher shear rates, the blood viscosity is determined primarily by RBC deformability. However, inducing RBC aggregation in a microfluidic channel at constant shearing blood flow controlled using a syringe pump is difficult. That is, several challenges (i.e., low flow rate of syringe pump or microfluidic channel dimension) make it difficult to set sufficiently low shear rates (i.e., $\dot{\gamma \;}$= 1–10 s^−1^) [20] to induce RBC aggregation in a microfluidic channel. Therefore, turning off the syringe pump or flow source, rather than decreasing the flow rate, is more effective [21,22]. Periodic on–off blood flow patterns are preferred to effectively obtain the biomechanical properties of blood [5,23]. Lee et al. suggested a blood-flow switching mechanism in a bridge-shaped fluidic channel. When the blood flow reverses in the bridge channel, the blood viscosity can be estimated using the flow rate ratio of the two fluids. Additionally, by changing the flow rate of blood suddenly and using the microscopic blood image intensity selected within the bridge channel (*A*), the time constant (*λ*) is obtained by obtaining a curve fitting of *A*(*t*) = *A*_0_ + *A*_1_ exp (−*t*/*λ*) [24]. Franke et al. suggested surface acoustic wave for extracting relation time of single RBCs [25]. Tiley et al. reported that surface area-to-volume ratio is major determinant of RBC passage in capillary vessel [26]. To obtain the RBC aggregation index (AI), the outlet of one channel filled with blood is closed periodically. The AI is estimated from the intensity of the blood flow image during the turn-off blood flow period [23]. More recently, Watanabe et al. suggested hematocrit-corrected aggregation index for comparative study of erythrocyte sedimentation rate [27]. Kakei et al. suggested an electrical impedance spectroscopy for monitoring RBC sedimentation in T-shaped container [28]. Elbuken et al. measured two haemorheological properties (i.e., *λ* and AI) in the capillary tube by analysing the blood–air interface and blood image intensity after turning off the pump [5]. Using variations of the optical transmitted signal (*I*), *λ* was calculated by conducting a nonlinear curve fitting of *I*(*t*) = *I*_0_ + *I*_1_ exp (−*t*/*λ*) [24]. They assumed that the time constant remained unchanged under transient blood flow. That is, a curve-fitting procedure was used to extract the time constant by analysing the blood velocity or interface [23,29]. However, as the time constant contributes to gradually decreasing transient blood flow, the blood flow does not stop immediately. This makes it difficult to induce RBC aggregation [30]. Therefore, the AI is significantly influenced by the time constant. However, unlike in previous studies, we expected that the time constant varies continuously under transient blood flow. Thus, the variation in the time constant under a transient blood flow must be extracted. Next, according to the well-known Maxwell model (i.e., time constant = viscosity/elasticity) [31], blood viscosity has a strong influence on the time constant. When the blood flow changes over time, blood viscosity cannot be obtained. If the correlation between blood viscosity and the time constant is validated, the time constant can be used effectively as an alternative property under dynamically varying blood flow conditions. Finally, for a quantitative comparison, three biomechanical properties of blood (i.e., blood viscosity, time constant, and RBC aggregation index) should be measured simultaneously under the square-wave profile of blood flows.

In this study, three haemorheological properties (i.e., viscosity, time constant, and RBC aggregation index) were obtained periodically by analysing blood flow, which was set to a square-wave profile. Based on the coflowing channel adapted for the measurement of blood viscosity [32], blood was injected periodically into a microfluidic device by turning the syringe pump on and off. To quantify blood viscosity, we supplied a reference fluid at a constant flow rate. The governing equation of the microfluidic system was derived using discrete fluidic circuit elements (i.e., flow rate, fluidic resistance, and compliance). Based on the governing equation, two properties (blood viscosity and time constant) were calculated sequentially for steady blood flow and transient blood flow. Unlike the previous studies, the present method does not assume that blood behaves as Newtonian fluid, and time constant remains constant under transient blood flow. RBC aggregation was then obtained by analysing the image intensity of microscopic blood images at stasis. Using glycerin solution (30%) as the test fluid, the contributions of the period (*T*), flow rate (Q), and air cavity to the time constant were evaluated quantitatively. The present method was then adopted to quantify the effect of Hct and RBC deformability on blood viscosity and the time constant. Finally, three mechanical properties of blood were measured with respect to several types of aggregation-elevated blood. We observed that the time constant exhibited consistent linear trends with respect to the blood viscosity. In addition, it exhibited reciprocal variations with elapsed time when compared with AI.

## 2. Materials and Methods

### 2.1. Experimental Setup, Flow Rate Setting, and Microscopic Image Acquisition

To effectively quantify the three haemorheological properties, we set up the experimental setup using a microfluidic device to guide blood flow, two syringe pumps to supply two fluids, and an image acquisition system to capture microscopic images of blood flows.

**Figure 1:**
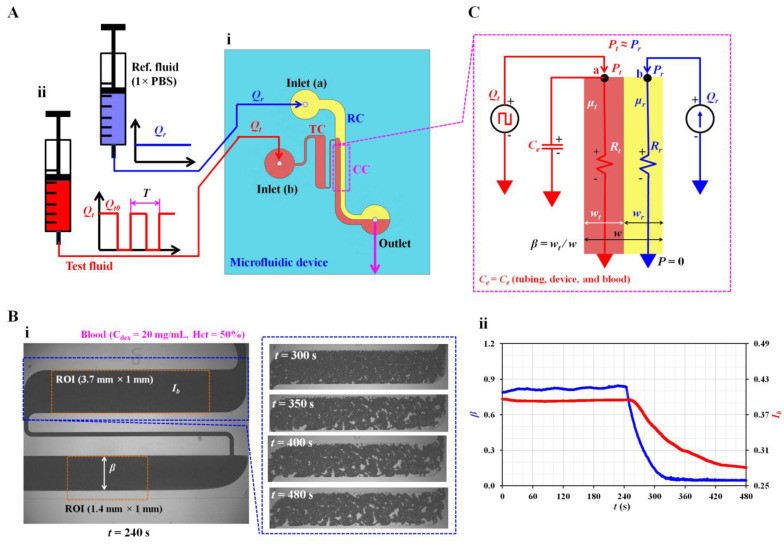
Proposed method for quantifying three haemorheological properties by analysing dynamic varying blood flow. (**A**) Schematic of the experimental setup, including a microfluidic device and two syringe pumps. (**i**) Microfluidic device with two inlets (a, b), an outlet, a reference fluid channel (RC), a test fluid channel (TC), and a coflowing channel (CC). (**ii**) Syringe pumps to supply test and reference fluids, respectively. Flow rate of reference fluid set to a constant value of *Q_r_*. Flow rate of test fluid (*Q_t_*) set to square-wave profile (i.e., maximum flow rate: *Q_t0_*, minimum flow rate: 0, period: *T*). (**B**) Quantification of microscopic image intensity of blood flow and interface in the coflowing channel. (**i**) Quantification of microscopic image intensity (*I_b_*) and interface (*β*). The right side panels show the variations in microscopic blood captured at a specific time (*t*) (*t* = 300, 350, 400, and 480 s) after the syringe pump was turned off. (**ii**) Temporal variations in *I_b_* and *β*. (**C**) Discrete fluidic circuit model to obtain blood viscosity and time constant in the coflowing channel.

Referring to a microfluidic device reported in a previous study [32], a coflowing channel was adopted in the present method to measure the viscosity and time constant, and a straight-wide channel was used to monitor the microscopic image intensity of blood flow. Blood flow in both channels was sufficiently visualised within the field of view (i.e., 4× objective lens [NA = 0.1]). As shown in Figure 1A-i the microfluidic device consisted of two inlets (a, b) to supply two fluids, an outlet to discard fluids from the device, a reference fluid channel (RC), a test fluid channel (TC), and a coflowing channel (CC). Here, the reference fluid (1 × phosphate-buffered saline [PBS]) and test fluid were passed through the reference fluid channel (width = 1000 µm) and test fluid channel (width = 1000 µm), respectively. Both fluids flowed in parallel to the coflowing channel (width = 1000 µm, length = 3500 µm). The channel depth (*h*) of the microfluidic device was set to *h* = 50 µm.

Using a soft lithography technique, a polydimethylsiloxane (PDMS, Sylgard 184, Dow Corning, Midland, MI, USA) block was replicated from a four-inch silicon master mould. Two inlets and an outlet were punched using a biopsy punch (outer diameter 0.75 mm). After the surface treatment with an oxygen plasma machine, a microfluidic device was fabricated by attaching a PDMS block to a glass slide. The microfluidic device was exposed to 120 °C for 10 min to maintain strong bonding between the components.

The microfluidic device was positioned on an inverted optical microscope (IX53, Olympus, Tokyo, Japan). Two types of identical polyethylene tubing (inner diameter = 0.25 mm, length = 400 mm) were inserted into each inlet (a, b). The third polyethylene tubing (inner diameter = 0.25 mm, length = 300 mm) was fitted to the outlet. To expel air in all channels of the microfluidic device and avoid non-specific adhesion of plasma proteins to the PDMS surface, we supplied bovine serum albumin (1 mg/mL) throughout the outlet. After 10 min, 1 × PBS was supplied through the tubing connected to the outlet. Two disposable syringes (~1 mL) were filled with the test and reference fluids. In this study, 1 × PBS was selected for clear interface due to difference in refractive index, especially in the coflowing channel. As shown in Figure 1A-ii, individual syringes were installed on each syringe pump (NeMESYS, Cetoni GmbH, Germany). The flow rate of the reference fluid was set as a constant (*Q_r_*). To measure haemorheological properties effectively, we set the flow rate of blood to the square-wave profile (i.e., maximum flow rate: *Q_t_*_0_, minimum flow rate: 0; period: *T*). In other words, blood viscosity was obtained under a constant flow rate of *Q_t_*_0_. The time constant was quantified by changing the flow rate from *Q_t_*_0_ to 0 (i.e., transient blood flow). RBC aggregation was measured at stasis (*Q_t_* = 0).

Sequential microscopic images of blood flow in the test channel and the interface in the coflowing channel were captured and recorded using a high-speed camera (FASTCAM MINI, Photron, Tokyo, Japan). The camera was set at 500 fps. Microscopic images of blood were captured sequentially in intervals of 0.5 s.

### 2.2. Digital Image Processing to Quantify Interface and Microscopic Image Intensity

The present method required two parameters related to blood flow: the interface in the coflowing channel and microscopic image intensity of blood flow in the test channel. As shown in Figure 1B-i, using a microscopic image captured with the camera, two parameters were obtained using digital image processing with MATLAB 2022a (MathWorks, Natick, MA, USA). Blood (Hct = 50%) was prepared by adding normal RBCs to dextran (20 mg/mL). It was supplied to a microfluidic channel with a square-wave profile (i.e., *Q_t0_* = 1 mL/h and *T*= 480 s). 1 × PBS was used as the reference fluid and supplied at constant flow rate of *Q_r_* = 1 mL/h. As shown in the left panel, a microscopic image was captured at *t* = 240 s. To obtain the interface in the coflowing channel, a specific region of interest (ROI, 1.4 mm × 1 mm) was selected downstream from the junction of the two fluids. After converting the grayscale microscopic image into a binary image, the averaged interface (*β*) was obtained by averaging the interfaces distributed over the ROI. In contrast, the right panel shows microscopic images captured at specific times (*t*) (*t* = 300, 350, 400, and 480 s). RBC aggregation began to occur after the syringe pump was turned off (i.e., *Q_t_* = 0) at *t* = 240 s. RBC aggregation contributed to increasing RBC-free space and varying image intensity. To quantify RBC aggregation at stasis, we selected a specific ROI (3.7 mm × 1 mm) within the test channel. The microscopic image intensity was obtained over time. The microscopic image intensity (*I_b_*) was obtained by averaging the intensity distributed over the ROI. The temporal variations in *β* and *I_b_* were obtained from the two image analysis procedures during a single period, as shown in Figure 1B-ii.

Finally, to monitor variations in blood flow in the test channel, we obtained velocity fields of blood flow using micro-particle image velocimetry (PIV) [33]. As shown in Figure 1B-i, a specific ROI (3.7 mm × 1 mm) was selected within the test channel. The average velocity (<*U_b_*>) was obtained by averaging the velocity fields distributed over the ROI. The blood flow rate was estimated as *Q_b_* =*A_c_* × <*U_b_*> by multiplying the cross-sectional area of the test fluid channel (*A_c_*) by the averaged velocity (<*U_b_*>).

### 2.3. Mathematical Formula of Three Haemorheological Properties

First, the mathematical formula of blood biomechanical properties (i.e., blood viscosity and time constant) was derived from a governing equation developed using discrete fluidic circuit elements. According to previous studies, fluid viscosity contributes to varying interfaces in the coflowing channel [9,32,34,35]. The interface in the coflowing channel was determined by the blood viscosity under a constant shearing blood flow. Additionally, the time constant was obtained by analysing the variations in the interface under transient blood flow. As shown in Figure 1C, a mathematical model of the two fluids in the coflowing channel was constructed using discrete circuit elements. The flow rates of the reference and test fluids were denoted as *Q_r_* and *Q_t_*. The reference fluid was supplied at a constant flow rate. However, the test fluid was supplied with a square-wave profile. Based on the viscosity ratio of the two fluids (i.e., *µ_t_*/*µ_r_*, where *µ_t_* is the viscosity of the test fluid, and is *µ_r_* the viscosity of the reference fluid), the corresponding width of each fluid was denoted as *w_r_* (reference fluid) and *w_t_* (test fluid) in the coflowing channel. The interface of the test fluid was denoted as the normalised width of the test fluid (i.e., *β* = *w_t_*/[*w_r_* + *w_t_*]). For a simple mathematical representation, the frictional losses of the two fluids were modelled as fluidic resistances (i.e., *R_r_* for the reference fluid and *R_t_* for the test fluid). To compensate for the approximation error caused by a simple mathematical model for real fluid flow [23,36], we measured the correction factor of fluidic resistance (α*_R_*) experimentally by relocating the interface (*β*) within both walls of the coflowing channel (i.e., α*_R_* = α*_R_* [*β*], 0 < *β* <1). Without the correction factor, the viscosity exhibited a significant error when the interface moved from the centre to both walls [9]. The contribution of flexible elements such as polyethylene tubing, microfluidic device, and blood was represented as an equivalent compliance element (i.e., *C_e_*). In the coflowing channel, the corresponding pressure of each fluid was denoted as *P_r_* (reference fluid stream) and *P_t_* (test fluid stream) at a distance (*L*) from the outlet. For convenience, the outlet was set to zero pressure (i.e., *P* = 0) and denoted as ▼ (i.e., ‘common ground’). As both fluids flowed in straight and parallel channels, they had identical pressures (i.e., *P_r_* = *P_t_*) at a specific distance. For the reference fluid stream, using the mass conservation law at point (a), Equation (1) was derived:

(1) $$\frac{P_{r}}{R_{r}}=Q_{r}$$

In addition, for the test fluid stream, using the mass conservation law at point (b), the following equation was derived

(2) $$C_{e}\frac{d}{dt}\left(P_{t}\right)+\alpha_{R}\left(\beta \right)\frac{P_{t}}{R_{t}}=Q_{t}$$

In Equation (2), *α_R_* (*β*) is multiplied by $\frac{P_{t}}{R_{t}}$ because it is related to the fluidic resistance. Based on the same pressure condition (i.e., *P_r_* = *P_t_*), substituting Equation (1) into Equation (2) yields:

(3) $$C_{e}\frac{d}{dt}\left(R_{r}\right)+\alpha_{R}\left(\beta \right)\frac{R_{r}}{R_{t}}=\frac{Q_{t}}{Q_{r}}$$

Each fluid stream was assumed to flow through two channels separated by a virtual wall (or interface). That is, a single coflowing channel was divided into two types of channels (i.e., reference and test fluid streams). Each separated channel was assumed to be rectangular. Under pressure-driven flow condition [37], pressure drop (Δ*P*) was expressed as Δ*P* = *R* × *Q*. Here, *R* and *Q* denoted fluidic resistance and flow rate, respectively. For a rectangular channel with a low aspect ratio (i.e., depth/width = 50/1000) [32], the fluidic resistance (*R*) of the fluid stream (i.e., *µ*: viscosity) in the rectangular channel is given as $R=\frac{12\;\mu \;L}{w\;h^{3}}$. Here, the corresponding fluidic resistance of each fluid was derived as $R_{r}=\frac{12\;\mu_{r}\;L}{\left(1-\beta \right)w\;h^{3}}$ (reference fluid stream) and $R_{t}=\frac{12\;\mu_{t}\;L}{\beta \;w\;h^{3}}$ (test fluid stream). By inserting two fluidic resistances into Equation (3), the following equation can be derived:

(4) $$\lambda_{t}\frac{d}{dt}\left(\frac{1}{1-\beta }\right)+\alpha_{R}\left(\beta \right)\frac{\beta }{1-\beta }=\left(\frac{Q_{t}}{Q_{r}}\right)\times \left(\frac{\mu_{t}}{\mu_{r}}\right)$$

In Equation (4), the time constant (*λ_t_*) is expressed as *λ_t_* = $\left(\frac{12\;\mu_{t\;L}}{w\;h^{3}}\right)$ × *C_e_*. That is, it is influenced by the viscosity of the test fluid and the equivalent compliance. Because the viscosity of the test fluid was measured under a constant shearing flow condition, the flow rate of the test fluid was set to *Q_t_* = *Q_t_*_0_. The first term in Equation (4) was set to zero (i.e., $\lambda_{t}\frac{d}{dt}\left[\frac{1}{1-\beta }\right]=0)$. The viscosity of the test fluid was then derived as:

(5) $$\mu_{t}=\mu_{r}\times \frac{\beta }{1-\beta }\times \left(\frac{Q_{r}}{Q_{t0}}\right)\times \alpha_{R}\left(\beta \right)$$

For Equation (5), the correction factor (*α_R_*) was obtained in advance using experiments. When the two fluids were set to the same flow rate (i.e., *Q_r_* = *Q_t_*_0_), the viscosity of the test fluid was obtained from the information of the interface (*β*) (i.e.,$\;\mu_{t}=\mu_{r}\times \frac{\beta }{1-\beta }\times \alpha_{R}\;[\beta$]). In contrast, by setting flow rate of test fluid to zero (i.e., *Q_t_* = 0), Equation (4) became

(6) $$\lambda_{t}\frac{d}{dt}\left(\frac{1}{1-\beta }\right)+\alpha_{R}\left(\beta \right)\times \left(\frac{\beta }{1-\beta }\right)=0$$

Here, the time constant (*λ_t_*) was assumed to vary with respect to *β*. Because *β* varied over time, *λ_t_* had a function of time (i.e., *λ_t_* = *λ_t_* [*t*]). At a certain time (*t* = *t_i_*), the first derivative term in Equation (6) was calculated in terms of *β* (*t_i−_*_1_) and *β* (*t_i+_*_1_) as follows:

(7) $$\lambda_{t}\frac{d}{dt}\left(\frac{1}{1-\beta }\right)=\lambda_{t}\left(t_{i}\right)\times \left(\frac{1}{t_{i+1}-t_{i-1}}\right)\times \left(\frac{1}{1-\beta \left[t_{i+1}\right]}-\frac{1}{1-\beta \left[t_{i-1}\right]}\right)$$

In addition, the second term in Equation (6) was evaluated using *β* (*t_i_*) as

(8) $$\alpha_{R}\left(\beta \right)\times \left(\frac{\beta }{1-\beta }\right)=\alpha_{R}\left(\beta \left[t_{i}\right]\right)\times \left(\frac{\beta \left[t_{i}\right]}{1-\beta \left[t_{i}\right]}\right)$$

By substituting Equations (7) and (8) into Equation (6), the formula for the time constant (*λ_t_*[*t_i_*]) was derived as

(9) $$\lambda_{t}\left(t_{i}\right)=-\left\{\alpha_{R}\left(\beta \left[t_{i}\right]\right)\times \left(\frac{\beta \left[t_{i}\right]}{1-\beta \left[t_{i}\right]}\right)\right\}/\left\{\left(\frac{1}{t_{i+1}-t_{i-1}}\right)\times \left(\frac{1}{1-\beta \left[t_{i+1}\right]}-\frac{1}{1-\beta \left[t_{i-1}\right]}\right)\right\}$$

Based on Equation (9), *λ_t_* was expressed as a function of the interface (*β*). Unlike in previous studies [5,30,38,39], the present method does not assume that the time constant remains unchanged with respect to the interface. Thus, based on Equation (9), variations in *λ_t_* were obtained with respect to the interface under transient blood flow conditions (i.e., *λ_t_* = *λ_t_* [*β*]).

From the mathematical model derived in this study, the blood viscosity and time constant of blood could be obtained using Equations (5) and (9), respectively.

**Figure A1:**
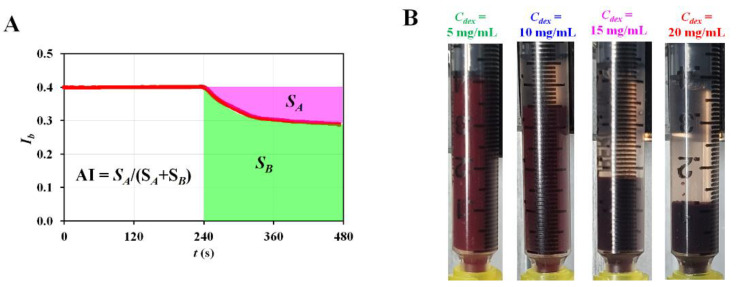
Quantification of RBC aggregation with aggregation index (AI). (**A**) Formula of AI by analysing temporal variations in *I_b_*. The AI was defined as AI = S*_A_*/(S*_A_* + S*_B_*). (**B**) Snapshots showing RBC sedimentation in the driving syringe captured at the end of experiments (*t* = 1680 s) with respect to *C_dex_*.

Second, based on a previous study, the RBC aggregation index (AI) was obtained at stasis by analysing the microscopic image intensity of blood in the test channel. As shown in Figure 1B-ii and Figure A1A (Appendix A), two factors (S*_A_* and S*_B_*) were calculated by analysing the temporal variations in *I_b_* from *t* = 240 s to *t* = 480 s (i.e., the turn-off period of the blood flow rate). The AI was then estimated as AI = S*_A_*/(S*_A_* + S*_B_*) [40,41,42]. Thus, the variations in AI were obtained with the elapsed time of every period.

### 2.4. Blood Preparation to Quantify Haemorheological Properties

The Gwangju–Chonnam Blood Bank (Gwangju, Republic of Korea) provided a concentrated RBC bag (~320 mL). The sample was stored in a refrigerator before the experiment. Based on the washing protocol reported in a previous study [39], normal RBCs were collected from concentrated RBCs. Suspended blood was then prepared by adding normal or hardened RBCs to various types of diluents, such as 1 × PBS and dextran solution. To adjust the degree of RBC rigidity, we prepared two different concentrations of diluted glutaraldehyde (GA) (*C_GA_* = 0.1% and 0.25%) by adding GA (Grade II, 25% in H20, Sigma-Aldrich, St. Louis, MO, USA) to 1 × PBS. Normal RBCs were exposed to each concentration of diluted GA. After 10 min, the hardened RBCs were collected by washing. In addition, four dextran solutions (*C_dex_* = 5, 10, 15, and 20 mg/mL) were prepared by dissolving dextran powder (*Leuconostoc* spp., MW = 450–650 kDa; Sigma-Aldrich, St. Louis, MO, USA) in 1 × PBS.

## 3. Results and Discussion

### 3.1. Quantification of Correction Factor and Rheological Properties of Glycerin

**Figure 2:**
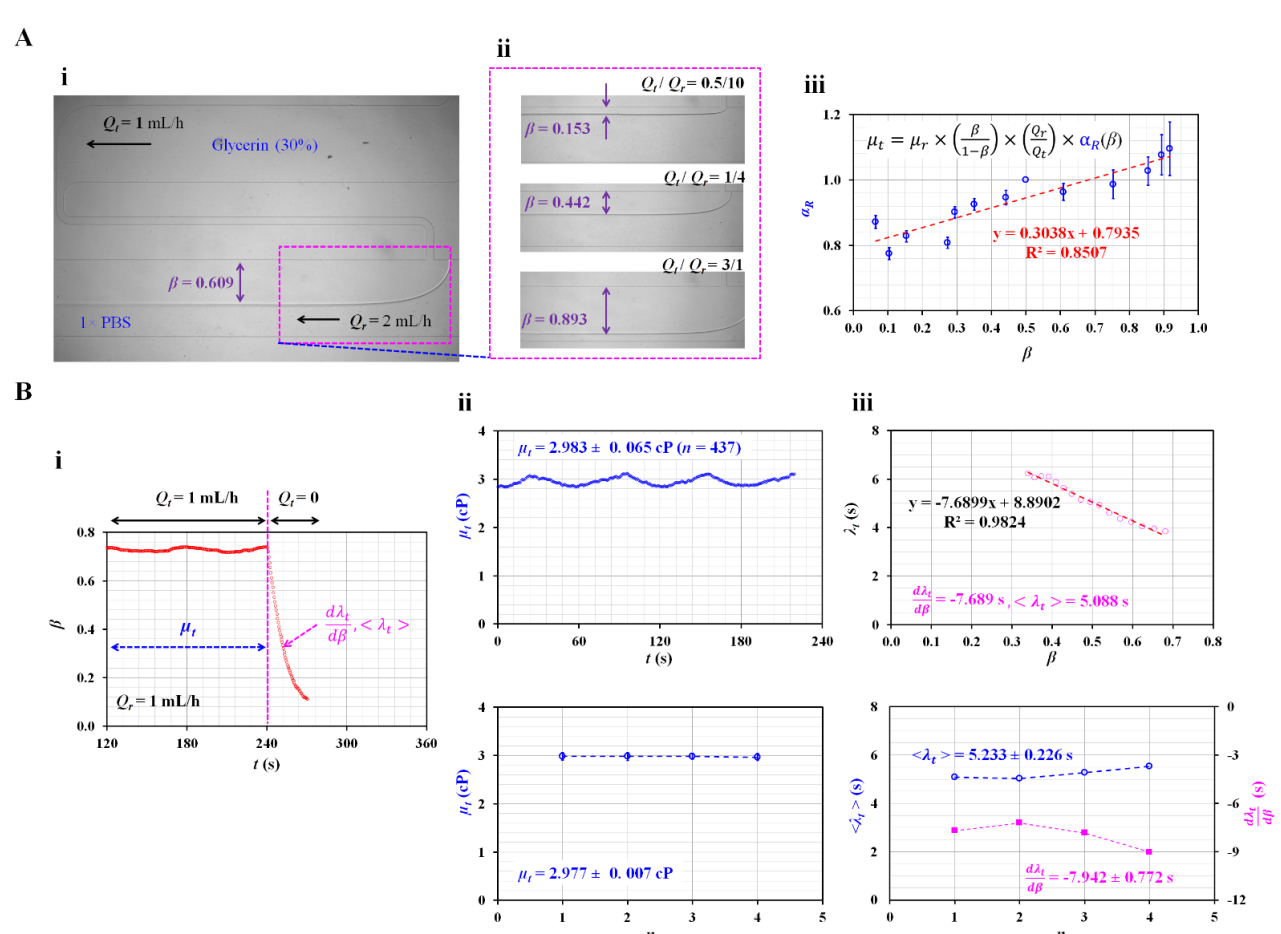
Quantification of the correction factor (*α_R_*) in the governing equation and determination of viscosity and time constant of glycerin (30%) as the test fluid. (**A**) Quantification of the correction factor (*α_R_*) as a linear expression of the interface in the coflowing channel. (**i**) Microscopic image of the interface in the coflowing channel. The arrow (‘←’) denoted flow direction of glycerin (30%) and 1× PBS. (**ii**) Variations in *β* with respect to flow rate ratio (*Q_t_*/*Q_r_*). (**iii**) Variations in correction factor (*α_R_*) with respect to *β*. (**B**) Measurement of viscosity (*µ_t_*) and time constant (*λ_t_*) under square-wave flow rate of the test fluid. (**i**) Viscosity and time constant were obtained at steady and transient flows, respectively. Linear slope (*dλ_t_*/*dβ*) and averaged time constant (<*λ_t_*>) were selected to quantify the dynamic variation in the test fluid. (**ii**) Temporal variations in *µ_t_* and variations of *µ_t_* over four periods (*n* = 4). The viscosity of glycerin (30%) was obtained as *µ_t_* = 2.977 ± 0.007 cP. (**iii**) Variations in *λ_t_* with respect to *β*, and variations in *dλ_t_*/*dβ* and <*λ_t_*> over four periods (*n* = 4). During four consecutive periods (*n* = 4), *dλ_t_*/*dβ* and <*λ_t_*> were obtained as –7.942 ± 0.772 s and 5.233 ± 0.226 s, respectively.

To measure fluid viscosity using Equation (5), we must determine the correction factor (*α_R_*) in advance. According to a previous study [23], the correction factor is influenced by several factors such as the aspect ratio (i.e., depth/width), viscosity of the reference fluid, and interface. In this study, 1 × PBS was used as the reference fluid. The channel dimensions (width and depth) were changed. As the channel dimension and reference fluid were changed, we had to obtain *α_R_* as a function of the interface (*β*). For convenience, glycerin (30%) was selected as the test fluid because it has very similar viscosity values as blood. Figure 2A-i shows a microscopic image of the interface in the coflowing channel. All experimental results were expressed as the mean ± standard deviation. *β* was obtained as 0.609 ± 0.006 at a constant flow rate of *Q_r_* = 2 mL/h and *Q_t_* = 1 mL/h. According to Equation (5), the interface (*β*) could be relocated by varying the flow-rate ratio (*Q_t_*/*Q_r_*). Figure 2A-ii shows the variations in *β* with respect to the flow rate ratio (*Q_t_*/*Q_r_*). The corresponding *β* of each flow-rate ratio was obtained as *β* = 0.153 ± 0.003 (*Q_t_*/*Q_r_* = 0.5/10), *β* = 0.442 ± 0.006 (*Q_t_*/*Q_r_* = 1/4), and *β* = 0.893 ± 0.005 (*Q_t_*/*Q_r_* = 3/1). By substituting the variations in *β* with respect to *Q_t_*/*Q_r_* into Equation (5), the correction factor (*α_R_*) was obtained as a function of *β*. According to the empirical formula reported in a previous study [43], the viscosity of the test fluid was set as *µ_t_* = 3 cP. As shown in Figure 2A-iii, the relation between the correction factor (*α_R_*) and interface (*β*) was verified using an XY plot (X axis: *β* and Y axis: *α_R_*). Using linear regression analysis, α*_R_* was obtained as *α_R_* = 0.3038 *β* + 0.7935. Because the coefficient of linear regression had a sufficiently high value of R^2^ = 0.8507, the linear regression formula could be considered statistically significant. Thus, the viscosity of the test fluid can be measured consistently using Equation (5) if the interface is obtained at a specific flow rate ratio of the two fluids.

Next, using Equations (5) and (9), the viscosity (*µ_t_*) and time constant (*λ_t_*) of the test fluid (glycerin [30%]) were measured under periodic on–off flow conditions. Instead of a constant flow rate, the test fluid was supplied with a square-wave profile (*Q_t_*_0_ = 1 mL/h and *T* = 480 s). The syringe pump for the reference fluid was set to *Q_r_* = 1 mL/h. As shown in Figure 2B-i, the viscosity of the test fluid was measured under steady flow conditions (*Q_t_* = 1 mL/h) for 240 s. Thereafter, by setting flow rate to zero (*Q_t_* = 0) from *t* = 240 s to *t* = 480 s, the time constant was measured at transient flow condition. As the time constant varied with *β*, the linear slope (*dλ_t_*/*dβ*) and averaged time constant (<*λ_t_*>) were selected as the representative parameters. The upper panel of Figure 2B-ii shows the temporal variations in *µ_t_* obtained under steady flow conditions. For a single period, *µ_t_* was expressed as 2.893 ± 0.065 (*n* = 437). The lower panel of Figure 2B-ii shows variations in *µ_t_* during the four periods (*n* = 4). The viscosity was measured consistently over four periods. The viscosity of glycerin (30%) was *µ_t_* = 2.977 ± 0.007 cP. Compared with the reference viscosity of glycerin (30%), the normalised difference between the present method and the reference value was less than 1%. Thus, the present method can be used to measure the viscosity of the test fluid. Next, the variations in *λ_t_* were obtained by analysing *β* under transient flow conditions. The upper panel of Figure 2B-iii shows the variations in *λ_t_* with respect to *β*. The *λ_t_* tended to increase linearly from *β* = 0.7 to *β* = 0.3. That is, when the flow rate of the test fluid decreased over time, the time constant tended to gradually increase. The *λ_t_* was influenced by viscosity as well as equivalent compliance. Here, viscosity of glycerin (30%) remained unchanged because it behaved as Newtonian fluid. Thus, it was inferred that equivalent compliance contributed to changing the time constant linearly. To represent variations in *λ_t_* under transient flow conditions, we obtained two parameters (i.e., linear slope: $\frac{d\lambda_{t}}{d\beta }$ and averaged value: <*λ_t_*>) using linear regression analysis and calculating the arithmetic average. *β,* as a non-dimensional parameter, was related to the volume of the test fluid in the coflowing channel. $\frac{d\lambda_{t}}{d\beta }$ could be physically interpreted as the change in the time constant when the volume of the test fluid decreased. According to the linear regression analysis, the regression formula was *λ_t_* = −7.6899 *β* + 8.8902 (R^2^ = 0.9824). The linear slope was obtained as $\frac{d\lambda_{t}}{d\beta }$ = −7.6899 s. As *β* was a nondimensional parameter, the unit of the linear slope was expressed as time (i.e., s). The arithmetic average of *λ_t_* was obtained as <*λ_t_*> = 5.088 s. The lower panel of Figure 2B-iii shows the variations in *dλ_t_*/*dβ* and <*λ_t_*> over four periods (*n* = 4). The representative parameters remained constant throughout the study period. *dλ_t_*/*dβ* and <*λ_t_*> were then obtained as *dλ_t_*/*dβ* = −7.942 ± 0.772 s and <*λ_t_*> = 5.233 ± 0.226 s.

The experimental results indicated that the present method can accurately calculate the viscosity of glycerin (30%). In addition, the time constant varies linearly with respect to the interface. The *dλ_t_*/*dβ* and <*λ_t_*> values are calculated and measured with consistency.

### 3.2. Contributions of Flow Rate Conditions of Test Fluid to Time Constant

According to previous studies [23,30,38,39], based on the assumption that the time constant remained unchanged over the interface, the time constant was obtained by analysing the interface or fluid velocity and conducting a curve-fitting procedure. Previous studies reported that the flow conditions of the test fluid influence the time constant. In this study, as the test fluid was supplied with a square-wave profile, it was necessary to evaluate the effect of the square-wave flow rate on variations in the time constant. Here, the maximum flow rate (*Q_t_*_0_) and period (*T*) of the square-wave profile were selected as vital factors. Compared with previous studies, this study did not assume that the time constant remained unchanged over the interface. That is, it could vary over the interface. To evaluate the contribution of the flow rate to the time constant, we used glycerin (30%) as the test fluid instead of blood. Additionally, 1 × PBS was used as the reference fluid at a constant flow rate (*Q_r_* = 1 mL/h).

**Figure 3:**
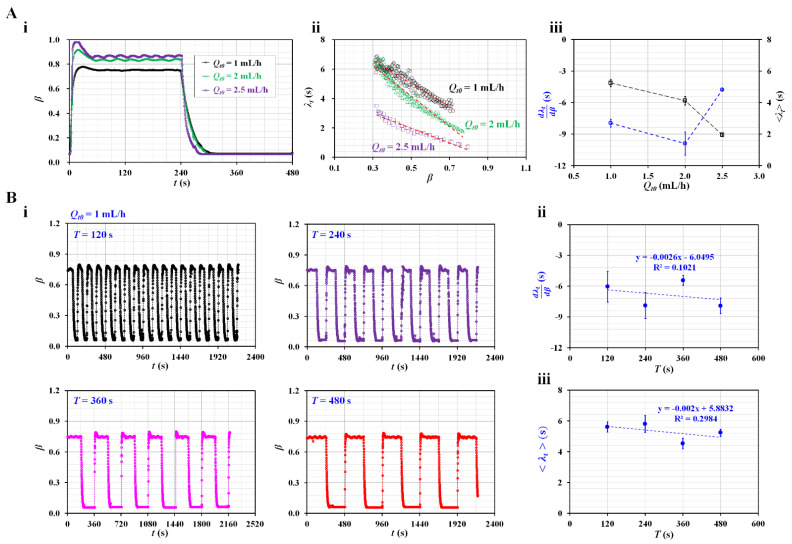
Contributions of flow rate condition of the test fluid (i.e., maximum flow rate and period) to the time constant. (**A**) Effect of the amplitude (*Q_t_*_0_) of the flow rate of the test fluid. The period was fixed at *T* = 480 s. (**i**) Temporal variations in *β* with respect to *Q_t0_* = 1, 2, and 2.5 mL/h. (**ii**) Variations in *λ_t_* with respect to *β* and *Q_t_*_0_. (**iii**) Variations in $\frac{d\lambda_{t}}{d\beta }$ and <*λ_t_*> with respect to Q*_t_*_0_. (**B**) Contribution of the period of the flow rate of the test fluid to the time constant. The amplitude of the flow rate of the test fluid was fixed at *Q_t_*_0_ = 1 mL/h. (**i**) Temporal variations in *β* with respect to *T* = 120, 240, 360, and 480 s. (**ii**) Variations in $\frac{d\lambda_{t}}{d\beta }$ with respect to *T*. (**iii**) Variations in <*λ_t_*> with respect to *T*.

As shown in Figure 3A, the effect of the maximum flow rate (*Q_t_*_0_) was evaluated by measuring the variation in the time constant (*λ_t_*) with respect to *Q_t_*_0_. For convenience, the period was set to a longer time of *T* = 480 s. Figure 3A-i shows temporal variations in *β* with respect to *Q_t_*_0_ = 1, 2, and 2.5 mL/h, during a single period of 480 s. For the steady flow condition, the interface tended to increase at a higher value of *Q_t_*_0_. Under the transient flow condition, *β* tended to decrease significantly at higher values of *Q_t_*_0_. Figure 3A-ii shows the variations in *λ_t_* with respect to *β* and *Q_t_*_0_. The time constant tended to increase linearly when the interface moved from *β* = 0.8 to *β* = 0.3. *λ_t_* tended to decrease at higher values of *Q_t_*_0_. As shown in Figure 3A-iii, using variations in *λ_t_* with respect to *β*, linear regression analysis was conducted to obtain $\frac{d\lambda_{t}}{d\beta }$ and <*λ_t_*> with respect to *Q_t_*_0_. The results indicated that <*λ_t_*> tended to decrease gradually with respect to *Q_t_*_0_. $\frac{d\lambda_{t}}{d\beta }$ did not exhibit consistent trends with respect to *Q_t_*_0_. According to a previous study [38], the time constant tended to decrease gradually by adjusting the flow rates from 1 to 2 mL/h. When compared with previous results, the <*λ_t_*> obtained using the present method exhibited similar trends. To obtain consistent results, we fixed *Q_t_*_0_. In the subsequent experiments, *Q_t_*_0_ was set to 1 mL/h (i.e., *Q_t_*_0_ = 1 mL/h).

Next, the effect of the period on the square-wave profile was evaluated by measuring the time constant with respect to each period. Figure 3B-i shows the temporal variations in *β* with respect to *T* = 120, 240, 360, and 480 s. Based on the temporal variations in *β* with respect to *T*, linear regression analysis was conducted to obtain $\frac{d\lambda_{t}}{d\beta }$ and <*λ_t_*>.

Figure 3B-ii shows the variations in $\frac{d\lambda_{t}}{d\beta }$ with respect to *T*. According to linear regression analysis, the coefficient of linear regression had a low value of R^2^ = 0.102. $\frac{d\lambda_{t}}{d\beta }$ did not have a linear relationship with *T*. Figure 3B-iii shows variations in <*λ_t_*> with respect to *T*. Linear regression analysis indicated that <*λ_t_*> did not have a linear relationship with *T* (i.e., R^2^ = 0.2984). A previous study reported that the time constant remained unchanged over a period ranging from *T* = 120 s to *T* = 240 s [23]. That is, the <*λ_t_*> obtained with the present method exhibited similar trends to the previous results. However, when the period was shorter, the flat value of *β* decreased significantly under steady flow conditions (i.e., *Q_t_* = *Q_t_*_0_). Therefore, a sufficiently flat range of *β* was required to obtain viscosity under steady flow rate conditions. For convenience, the period was set to a longer duration of *T* = 480 s during the subsequent experiments.

### 3.3. Effect of Air Cavity Secured in the Driving Syringe on the Time Constant

Compliance units, including air bubble [44,45,46,47] and flexible membrane [48,49] have been used to regulate unstable blood flow. However, the compliance effect contributes significantly to the delay in the dynamic response. The air cavity inside the driving syringe caused an increase in the time constant [30]. Thus, the higher value of the time constant hindered the measurement of RBC aggregation under specific off-period flow conditions. Based on previous studies, the air cavity was secured appropriately inside the driving syringe to distinctively increase the time constant under the current fluidic system. Here, two fluids (i.e., glycerin [30%] and blood) were selected as test fluids to quantify the effect of the air cavity (*V_air_*) secured inside the driving syringe on the variation of the time constant. An air cavity was secured above the test fluid in the driving syringe.

**Figure 4:**
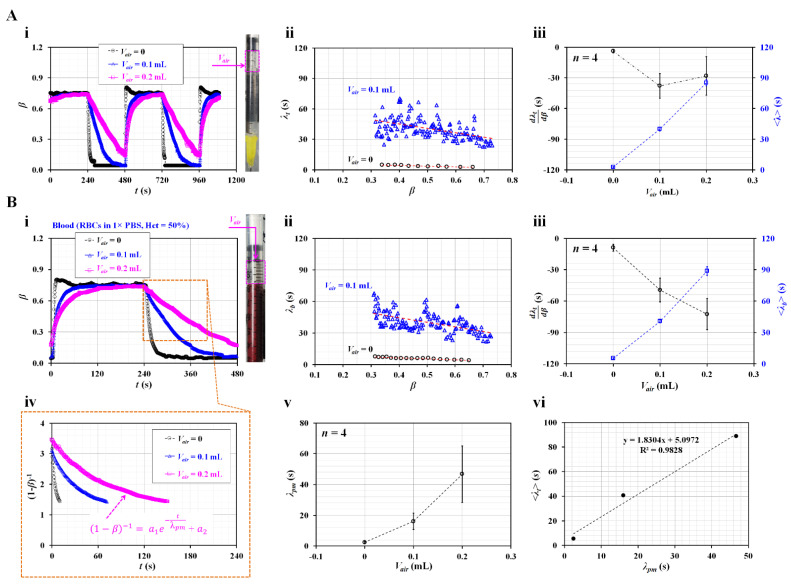
Contribution of air cavity secured inside the driving syringe (*V_air_*) to the time constant. (**A**) Contributions of *V_air_* to the time constant of glycerin (30%). (**i**) Temporal variations in *β* with respect to *V_air_* = 0, 0.1, and 0.2 mL. The right-hand panel shows the air cavity secured over glycerin (30%) inside the drying syringe. (**ii**) Variations in *λ_t_* with respect to *β* and *V_air_*. (**iii**) Variations in $\frac{d\lambda_{t}}{d\beta }$ and <*λ_t_*> with respect to *V_air_*. (**B**) Contribution of *V_air_* to the time constant of blood. Here, blood (Hct = 50%) was prepared by adding normal RBCs into 1 × PBS. (**i**) Temporal variations in *β* with respect to *V_air_* = 0, 0.1, and 0.2 mL. The right-hand panel shows the air cavity secured above blood inside the drying syringe. (**ii**) Variations in *λ_t_* with respect to *β* and *V_air_*. (**iii**) Variations in $\frac{d\lambda_{t}}{d\beta }$ and <*λ_t_*> with respect to *V_air_*. (**iv**) Temporal variations in (1−*β*)^−1^ with respect to *V_air_*. Here, the variation in (1−*β*)^−1^ was best fitted as (1−*β*)^−1^ = *a_1_* exp (–*t*/*λ_pm_*) + *a_2_*. (**v**) Variations in *λ_pm_* with respect to *V_air_*. (**vi**) Linear relationship between <*λ_t_*> (present method) and *λ_pm_* (previous method).

First, the driving syringe was filled with glycerin (30%) as the test fluid. As shown in the right-hand panel of Figure 4A-i, the air cavity was secured above the glycerin (30%) from *V_air_* = 0 to *V_air_* = 0.2 mL. Figure 4A-i shows the temporal variations in *β* with respect to *V_air_* = 0, 0.1, and 0.2 mL. The higher volume of the air cavity contributed to the gradually changing transient variations in *β* over time. The flat range of *β* decreased at higher values of the air cavity. At *V_air_* = 0.2 mL, when the syringe pump was turned on for 240 s, *β* increased gradually over time. The flat interval, *β*, is very short. The short interval of flat *β* made it difficult to obtain the viscosity during steady flow conditions. Figure 4A-ii shows the variations in *λ_t_* with respect to *β* and *V_air_*. When *V_air_* was changed from 0 to 0.1 mL, *λ_t_* increased significantly. As it had large scatters over *β*, the linear regression analysis did not guarantee a consistent linear relationship between *λ_t_* and *β*. Figure 4A-iii shows variations in $\frac{d\lambda_{t}}{d\beta }$ and <*λ_t_*> with respect to *V_air_*. Statistical data (i.e., mean and standard deviation) were obtained from four sets of $\frac{d\lambda_{t}}{d\beta }$ and <*λ_t_*> (*n* = 4). When *V_air_* set to 0.1 or 0.2 mL, $\frac{d\lambda_{t}}{d\beta }$ decreased largely when compared with no air cavity. However, it exhibited a large scatter. Thus, determining a consistent linear relationship between $\frac{d\lambda_{t}}{d\beta }$ and *V_air_* was difficult. <*λ_t_*> increased significantly with respect to the air cavity (i.e., <*λ_t_*> ~ *V_air_*). These results exhibited similar trends and the same order of time constants as those in a previous study [30].

Second, glycerin (30%), as the test fluid, was replaced by suspended blood. Blood samples (Hct = 50%) were prepared by adding normal RBCs to 1 × PBS. Figure 4B-i shows the temporal variations in *β* with respect to *V_air_* = 0, 0.1, and 0.2 mL. The right panel shows the air cavity secured over blood inside the drying syringe. Transient variations in *β* exhibited similar trends to those of *β* as shown in Figure 4A-i. Blood viscosity was obtained as *µ_b_* = 2.92 ± 0.02 during steady blood flow. That is, two fluids had very similar values of viscosity (i.e., *µ* = 2.98 ± 0.01 cP for glycerin [30%], and *µ* = 2.92 ± 0.02 cP for blood). Figure 4B-ii shows the variations in *λ_t_* with respect to *β* and *V_air_*. *λ_t_* exhibited similar trends with respect to *β* when compared with the time constant of glycerin (30%). When *V_air_* was set to 0.1 mL, *λ_t_* increased and exhibited large scatters compared with no air cavity. It tended to increase when the interface was moved from *β* = 0.7 to *β* = 0.3. Figure 4B-iii shows the variations in $\frac{d\lambda_{t}}{d\beta }$ and <*λ_t_*> with respect to *V_air_*. <*λ_t_*> tended to increase with respect to the air cavity. <*λ_t_*> of the blood was similar to that of glycerin (30%), as shown in Figure 4A-iii. We inferred that <*λ_t_*> was proportional to viscosity, and both fluids had similar viscosity values. $\frac{d\lambda_{t}}{d\beta }$ increased significantly when the air cavity was set to 0.1 mL. It did not exhibit significant difference between *V_air_* = 0.1 mL to *V_air_* = 0.2 mL. Furthermore, as shown in Figure 4A-ii,B-ii, *λ_b_* exhibited large scatters and did not have a consistent linear relationship with respect to *β*. Statistically, $\frac{d\lambda_{t}}{d\beta }$ did not provide meaningful information except *V_air_* = 0. As the two types of test fluids had similar viscosity values, they had similar trends of $\frac{d\lambda_{t}}{d\beta }$ and <*λ_t_*>. The air cavity contributed to varying the time constant rather than the test fluid.

Finally, for a quantitative comparison, the previous method was adopted to calculate the time constant of the blood. With the previous method [23], the time constant was assumed to be a single value during the transient variation in *β*. The time constant was obtained using a curve-fitting technique [30,38]. That is, based on the governing equation of Equation (4), *β* was replaced by (1−*β*)^−1^. Figure 4B-iv shows the temporal variations in (1−*β*)^−1^ with respect to *V_air_*. The large value of the air cavity caused to change (1−*β*)^−1^ gradually. The variations in (1−*β*)^−1^ were best fitted as (1−*β*)^−1^ = *a*_1_ exp (−*t*/*λ_pm_*) + *a*_2_. Here, *λ_pm_* denotes the time constant suggested by the previous method. By conducting nonlinear regression analysis with MATLAB 2022a, *λ_pm_* was obtained with respect to *V_air_*. Figure 4B-v shows the variations in *λ_pm_* with respect to *V_air_*. *λ_pm_* tended to increase and exhibited large scatter at large values of the air cavity. For comparison with <*λ_t_*> obtained using the present method, as shown in Figure 4B-vi, *λ_pm_* and <*λ_t_*> were plotted on the X axis and Y axis, respectively. As $\frac{d\lambda_{t}}{d\beta }$ did not present statistical significance with respect to the air cavity, <*λ_t_*> was selected for the present method. <*λ_t_*> was larger than *λ_pm_*. The linear relationship between the proposed and previous methods was obtained as <*λ_t_*> = 1.8304 *λ_pm_* + 5.0972 by conducting a linear regression analysis. As the coefficient of regression has a sufficiently high value of R^2^ = 0.9828, <*λ_t_*> showed comparable trends to *λ_pm_*. The results showed that the air cavity significantly contributed to increasing the time constant. Thus, it hindered the measurement of viscosity under steady flow conditions. Therefore, when the driving syringe was filled with the test fluid, the air cavity was not secured above the test fluid in the syringe.

### 3.4. Effect of Haematocrit on Viscosity as Well as Time Constant

Instead of glycerin (30%) (i.e., pure liquid), normal suspended blood was selected as the test fluid. To alter the haemorheological properties, each blood sample had different Hct levels. The haemorheological properties were measured under two blood flow conditions (i.e., constant flow rate and square-wave blood rate).

**Figure 5:**
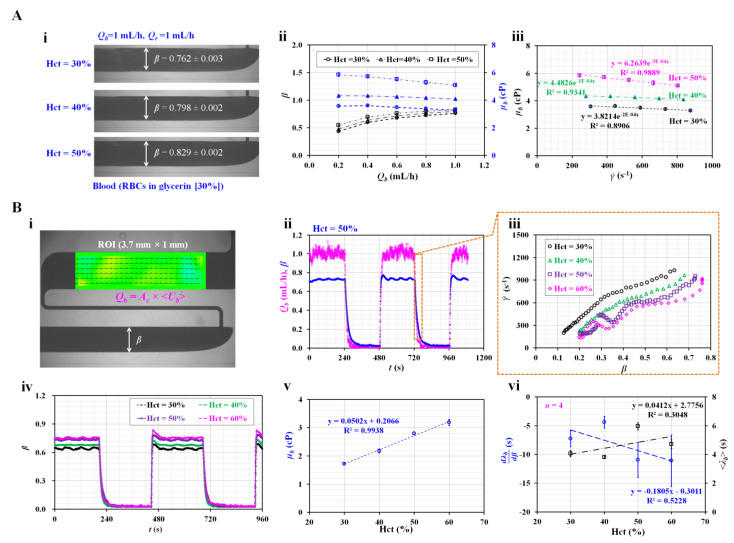
Contribution of Hct to haemorheological properties (i.e., viscosity and time constant) under steady flow rate and square-wave profile of blood flow. (**A**) Effect of Hct on blood viscosity under steady blood flow. (**i**) Variation in *β* with respect to Hct. (**ii**) Variation in *β* and *µ_b_* with respect to blood flow rate (i.e., *Q_b_* = 0.2, 0.4, 0.6, 0.8, and 1 mL/h) and Hct (i.e., Hct = 30%, 40%, and 50%). (**iii**) Variation in *µ_b_* with respect to shear rate and Hct. (**B**) Effect of Hct on the viscosity and time constant under square-wave profile of blood flow. (**i**) Quantification of flow rate and *β*. (**ii**) Temporal variations in *Q_b_* and *β* for blood (Hct = 50%). (**iii**) Variations in shear rate with respect to *β* and Hct = 30%, 40%, 50%, and 60%, under transient blood flow. (**iv**) Temporal variations in *β* with respect to Hct. (**v**) Variations in *µ_b_* with respect to Hct. (**vi**) Variations in $\frac{d\lambda_{t}}{d\beta }$ and <*λ_t_*> with respect to Hct.

First, the contribution of Hct to the blood viscosity was evaluated by measuring the blood viscosity with respect to three Hct levels (i.e., Hct = 30%, 40%, and 50%). As blood behaves as a non-Newtonian fluid, its viscosity decreases gradually with respect to the shear rate. To change the shear rate of the blood flow, we set the flow rate to *Q_b_* = 0.2, 0.4, 0.6, 0.8, and 1 mL/h. Based on previous studies [30,36], the corresponding shear rate of the bloodstream in the coflowing channel was estimated using the analytical formula of shear rate (i.e., $\dot{\gamma }=\frac{6\;Q_{b}}{\beta \;w\;h^{2}}$) in the coflowing channel [5]. As the width (*w*) and depth (*h*) were fixed, the shear rate of the blood flow depended on the interface (*β*) in the coflowing channel under a constant flow rate of *Q_b_*. To avoid RBC sedimentation inside the driving syringe, we prepared the blood by suspending normal RBCs in glycerin (30%) rather than 1 × PBS [50]. Figure 5A-i shows microscopic images of the two fluids in the coflowing channel with respect to the Hct level. Here, the two fluids were supplied at the same flow rate (*Q_b_* = *Q_r_* = 1 mL/h). The corresponding *β* of each Hct level was obtained as *β* = 0.762 ± 0.003 (Hct = 30%), *β* = 0.798 ± 0.002 (Hct = 40%), and *β* = 0.829 ± 0.002 (Hct = 50%). Figure 5A-ii shows the variations in *β* and *µ_b_* with respect to *Q_b_*. Higher Hct values contributed to increasing *β* and *µ_b_*. In addition, *µ_b_* tended to decrease with respect to *Q_b_*. In other words, blood behaved as a non-Newtonian fluid. The corresponding shear rate for each flow rate was estimated using the shear rate formula, i.e., words the shear rate was estimated by inserting *Q_b_* into $\dot{\gamma }$. The variation in the blood viscosity was replotted with respect to $\dot{\gamma }$. As shown in Figure 5A-iii, the blood viscosity tended to decrease gradually as $\dot{\gamma }$ ranged from 200 to 900 s^−1^. Glycerin (30%) did not contain plasma proteins. Additionally, RBC aggregation was not involved in the variation in blood viscosity at sufficiently high shear rates. Thus, RBC deformability and alignment decreased the blood viscosity in a power-law fashion (i.e., $\mu_{b}=\mu_{0}{\left(\dot{\gamma }\right)}^{n-1}$) [6,11,51]. According to the nonlinear regression analysis, the coefficient of each regression formula ranged from R^2^ = 0.8906 to R^2^ = 0.9889. The blood viscosity decreased according to the power law with respect to the shear rate. Furthermore, it increased significantly at higher Hct values.

Second, instead of a steady flow rate, the flow rate was set to a square-wave profile (i.e., *Q_t_*_0_ = 1 mL/h and *T* = 480 s). The blood viscosity was measured at a steady flow rate (*Q_b_* = 1 mL/h). The time constant of the blood flow was obtained under transient blood flow by setting the blood flow rate from *Q_b_* = 1 mL/h to *Q_b_* = 0. Here, the Hct of blood was adjusted to 30%, 40%, 50%, and 60% by suspending normal RBCs in 1 × PBS. Figure 5B-i shows the quantification of the blood flow rate (*Q_b_*) in the test fluid channel and the interface (*β*) in the coflowing channel. To estimate the variation in the shear rate under transient blood flow, we obtained variations in *Q_b_* with respect to time. Here, a micro-PIV technique was adopted to obtain the velocity fields of blood in the test fluid channel. After calculating the average velocity of blood (<*U_b_*>), the flow rate of blood (*Q_b_*) was obtained as *Q_b_* = *A_c_* × <*U_b_*>. Here, *A_c_* denotes the cross-sectional area. Figure 5B-ii shows the temporal variations in *Q_b_* and *β* for blood (Hct = 50%). Based on the temporal variations of *Q_b_* under transient blood flow, the shear rate of blood flow was estimated with respect to *Q_b_*. In addition, the variation in *β* was obtained at a specific time. Thus, we could create an XY plot (i.e., X axis: *β* and Y axis: $\dot{\gamma }$). As shown in Figure 5B-iii, the variations in the shear rate were obtained with respect to *β* and Hct. Lower values of the Hct had higher shear rates. The shear rate was varied from 150 to 1300 s^−1^. For convenience, the low threshold of *β* was set to 0.3 (i.e., *β* > 0.3). The shear rate was then estimated above 250 s^−1^. As shown in Figure 5A-iii, the blood behaved as a non-Newtonian flow. In this study, the viscosity or time constant was assumed to fluctuate over *β*. Unlike previous studies [23,38], we could obtain variations in the time constant with respect to *β* (i.e., *λ_b_* = *λ_b_* [*β*]). However, as the blood viscosity was obtained at a constant flow rate, obtaining variations in the blood viscosity under transient blood flow was impossible. Figure 5B-iv shows the temporal variations in *β* with respect to Hct. At a steady flow rate of 1 mL/h, *β* increased significantly with respect to Hct. Using Equation (5), the variation in *µ_b_* was obtained with respect to Hct. As shown in Figure 5B-v, according to the linear regression analysis, the blood viscosity was proportional to Hct (i.e., *µ_b_* = 0.0502 Hct + 0.2066, R^2^ = 0.9938). Under transient blood flow, the time constant was obtained by analysing *β* over time. As shown in Figure 5B-vi, variations in $\frac{d\lambda_{t}}{d\beta }$ and <*λ_t_*> were obtained with respect to Hct. $\frac{d\lambda_{t}}{d\beta }$ tended to decrease with respect to Hct. Additionally, <*λ_t_*> tended to increase with respect to Hct. As the time constant was proportional to the blood viscosity (i.e., *λ_b_* = $\left[\frac{12\;\mu_{b\;L}}{w\;h^{3}}\right]$ × *C_e_*), it was reasonable that <*λ_t_*> increased at higher Hct values. Under transient blood flow, we inferred that the blood viscosity or compliance (*C_e_*) caused an increase in the time constant with respect to Hct. However, regression analysis indicated that both $\frac{d\lambda_{t}}{d\beta }$ and <*λ_t_*> exhibited statistical significance with respect to Hct (i.e., R^2^ = 0.3048–0.5228). From the results, the blood viscosity could be selected to monitor variations in Hct effectively instead of the time constant.

### 3.5. Detection of Hardened RBCs with Viscosity and Time Constant

**Figure 6:**
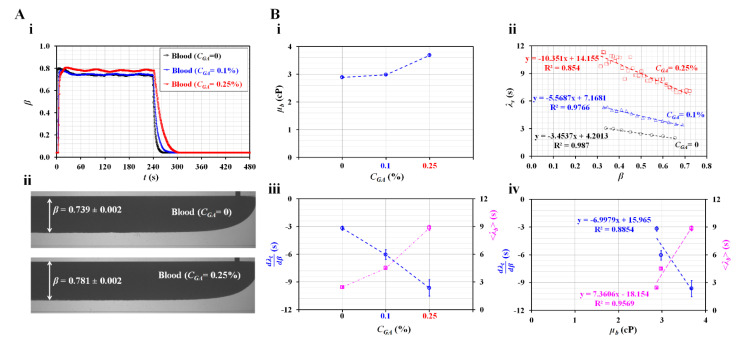
Quantification of haemorheological properties of hardened RBCs. (**A**) Variations in the interface with respect to concentration of GA solution. (**i**) Temporal variations in *β* with respect to the concentration of GA solution (*C_GA_* = 0, 0.1%, and 0.25%). (**ii**) Microscopic images of the interface with respect to *C_GA_*. (**B**) Quantification of viscosity and time constant of hardened RBCs. (**i**) Variations in *µ_b_* with respect to *C_GA_*. (**ii**) Variations in *λ_b_* with respect to *β* and *C_GA_*. (**iii**) Variations in $\frac{d\lambda_{t}}{d\beta }$ and <*λ_t_*> with respect to *C_GA_*. (**iv**) Variations in $\frac{d\lambda_{t}}{d\beta }$ and <*λ_t_*> with respect to *µ_b_.*

Instead of normal blood, hardened RBCs were prepared to detect their contribution of hardened RBCs to variations in rheological properties. Hardened blood (Hct = 50%) was prepared by adding chemically fixed RBC into 1 × PBS. Figure 6A-i shows the temporal variations in *β* with respect to the concentration of diluted glutaraldehyde (*C_GA_*) (*C_GA_* = 0, 0.1%, and 0.25%). Figure 6A-ii shows microscopic images for detecting the interface with respect to *C_GA_*. The corresponding *β* for each concentration of GA was obtained as *β* = 0.739 ± 0.002 (C_GA_ = 0) and *β* = 0.781 ± 0.002 (C_GA_ = 0.25%). For hardened RBCs with *C_GA_* = 0.25%, *β* increased at steady blood flow compared with control blood (*C_GA_* = 0). Under transient blood flow, the hardened blood contributed to a gradually changing *β* over time.

Figure 6B-i shows the variations in *µ_b_* with respect to *C_GA_*. When normal RBCs were exposed to higher concentrations of GA, the degree of RBC hardness increased significantly. Hardened RBCs contributed to increased blood viscosity. Figure 6B-ii shows the variations in the time constant (*λ_b_*) with respect to *β* and *C_GA_*. *λ_b_* increased linearly with decreasing *β*. According to the linear regression analysis, the corresponding formulas for each concentration of GA were obtained as *λ_b_* = −3.4537*β* + 4.2013 (C_GA_ = 0), *λ_b_* = −5.5687*β* + 7.1681 (*C_GA_* = 0.1%), and *λ_b_* = −10.351*β* + 14.155 (*C_GA_* = 0.25%). The coefficient of linear regression had a high value of R^2^ = 0.854–0.987. Figure 6B-iii shows the variations in $\frac{d\lambda_{t}}{d\beta }$ and <*λ_t_*> with respect to *C_GA_*. $\frac{d\lambda_{t}}{d\beta }$ decreased linearly with respect to *C_GA_*. In addition, <*λ_t_*> increased linearly with respect to C_GA_. Hardened RBCs contributed to increasing <*λ_t_*> and decreasing $\frac{d\lambda_{t}}{d\beta }$ when compared with normal RBCs. Figure 6B-iv shows the variations in $\frac{d\lambda_{t}}{d\beta }$ and <*λ_t_*> with respect to *µ_b_.* According to the linear regression analysis, the coefficient of linear regression had a sufficiently high value of R^2^ = 0.8854–0.9569. These results indicated that both $\frac{d\lambda_{t}}{d\beta }$ and <*λ_t_*> were proportional to *µ_b_*. Thus, hardened RBCs could be detected effectively in terms of either the blood viscosity or time constant.

Previous studies assumed that the time constant is independent of *β* (i.e., *λ_b_* = constant). It was then obtained by curve-fitting the averaged velocity in the main channel. Previous studies designed channels with significantly higher fluidic resistance using a low value of channel depth (i.e., depth = 10–20 µm) when compared with this study [38,39]. For hardened RBCs with GA ranging from 0.05% to 0.25%, the time gradually decreased at higher concentrations of GA. The hardened blood exhibited higher blood viscosity. According to Maxwell’s law (i.e., time constant = viscosity/elasticity) [24], the elasticity tended to increase for hardened RBCs. This is reasonable from a biophysical perspective. However, the time constants obtained in this study exhibited different trends with respect to the concentration of GA. Compared with previous studies, there was only a difference in the fluidic channel. Thus, we estimated that the difference in trends in the time constant resulted from the difference in the fluidic resistance. To effectively monitor variations in RBCs hardness under transient blood flow, we set a significantly higher fluidic resistance value. Nonetheless, the blood viscosity obtained using the present method exhibited sufficiently consistent trends when compared with previous studies. In addition, the parameters of the time constant (i.e., $\frac{d\lambda_{t}}{d\beta }$ and <*λ_t_*>) exhibited a linear relationship with the blood viscosity.

### 3.6. Detection of RBC Aggregation-Elevated Blood

RBC sedimentation in the driving syringe caused an increase in the Hct of the blood flows in the microfluidic channel [52]. The increase in the Hct contributed to a decrease in RBC aggregation. The Hct level changed continuously. This contributed to the varying mechanical properties of blood over time. According to previous studies, variations in RBC aggregation were monitored by analysing microscopic image intensity [40,50], optical transmitted intensity [22,42], and impedance [21,41,53,54] after sufficient blood flow was stopped. However, the blood viscosity was obtained under steady blood flow. The present method was employed to quantify variations in the three biomechanical properties of blood (i.e., viscosity, time constant, and AI) resulting from RBC sedimentation in the driving syringe. To gradually adjust the degree of RBC aggregation, we prepared suspended blood (Hct = 50%) by adding normal RBCs to four different concentrations of dextran (*C_dex_* = 5, 10, 15, and 20 mg/mL).

**Figure 7:**
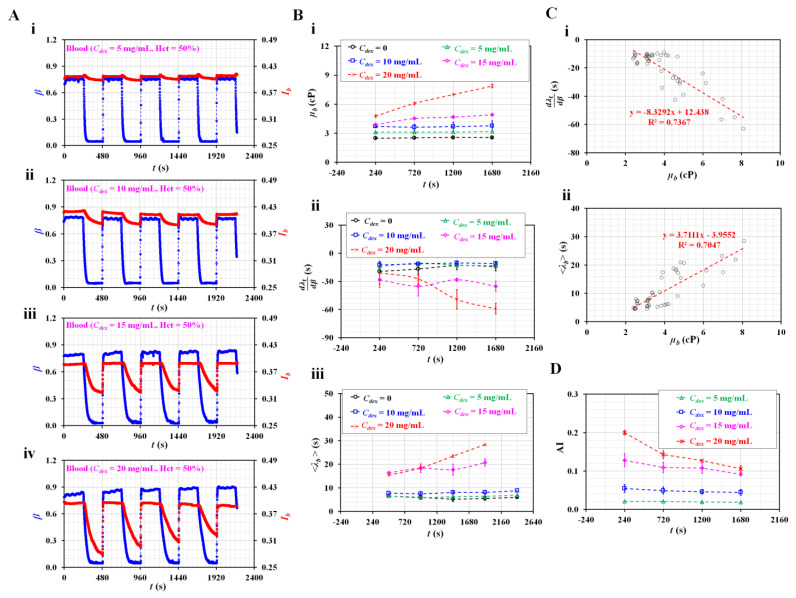
Quantification of haemorheological properties of RBC aggregation-induced bloods. (**A**) Temporal variations in interface (*β*) and image intensity of blood (*I_b_*) with respect to concentration of dextran (*C_dex_*) ((**i**) *C_dex_* = 5 mg/mL, (**ii**) *C_dex_* = 10 mg/mL, (**iii**) *C_dex_* = 15 mg/mL, and (**iv**) *C_dex_* = 20 mg/mL). (**B**) Variations in the haemorheological properties with respect to *C_dex_*. (**i**) Temporal variations in *µ_b_* with respect to *C_dex_*. (**ii**) Temporal variations in $\frac{d\lambda_{t}}{d\beta }$ with respect to *C_dex_*. (**iii**) Temporal variations in <*λ_t_*> with respect to *C_dex_*. (**C**) Linear relationship between blood viscosity and time constant for aggregation-enhanced bloods. (**i**) Relationship between $\frac{d\lambda_{t}}{d\beta }$ and *µ_b_*. (**ii**) Relationship between <*λ_t_*> and *µ_b_*. (**D**) Quantification of RBC aggregation using RBC aggregation index (AI) with respect to *C_dex_*.

Figure 7A shows the temporal variations in *β* and *I_b_* with respect to the concentration of dextran (*C_dex_*) ([i] *C_dex_* = 5 mg/mL, [ii] *C_dex_* = 10 mg/mL, [iii] *C_dex_* = 15 mg/mL, and [iv] *C_dex_* = 20 mg/mL). Under steady flow conditions, *β* tended to increase at higher dextran concentrations. Above *C_dex_* = 15 mg/mL, *β* tended to increase gradually over time. In addition, during the turn-off period of the blood flow rate, the variations in *I_b_* increased significantly at higher dextran solution concentrations. Variations in *I_b_* tended to decrease over consecutive periods. However, *I_b_* did not exhibit a significant difference at steady blood flow. Figure 7B-i shows the temporal variations in *µ_b_* with respect to *C_dex_*. The blood viscosity increased at higher concentrations of dextran. Dextran contributed to increasing blood viscosity. In addition, for suspended blood with a low concentration of dextran (*C_dex_* = 0, 5, and 10 mg/mL), blood viscosity remained unchanged over time. However, it increased significantly over time, particularly for suspended blood with higher concentrations of dextran (i.e., *C_dex_* = 15 and 20 mg/mL).

Figure 7B-ii shows the temporal variations in $\frac{d\lambda_{t}}{d\beta }$ with respect to *C_dex_*. $\frac{d\lambda_{t}}{d\beta }$ tended to decrease at higher dextran concentrations. At low dextran concentrations, the difference in $\frac{d\lambda_{t}}{d\beta }$ was minimal (i.e., *C_dex_* = 0, 5, and 10 mg/mL). However, $\frac{d\lambda_{t}}{d\beta }$ decreased significantly at higher dextran concentrations (*C_dex_* = 15 and 20 mg/mL). Figure 7B-iii shows the temporal variations in <*λ_t_*> with respect to *C_dex_*. <*λ_t_*> tended to increase at higher dextran concentrations. In addition, it increased significantly over time in blood at higher dextran concentrations (i.e., *C_dex_* = 15 and 20 mg/mL). However, <*λ_t_*> did not exhibit a significant difference with time at low dextran concentrations (i.e., *C_dex_* = 0, 5, and 20 mg/mL). Based on the experimental results shown in Figure 7B, the linear relationship between the blood viscosity (*µ_b_*) and time constant (i.e., $\frac{d\lambda_{t}}{d\beta }$, and <*λ_t_*>) was validated using a regression analysis. Figure 7C-i shows the XY plot to verify the linear relationship between $\frac{d\lambda_{t}}{d\beta }$ and *µ_b_*. According to the linear regression analysis, the regression formula was obtained as $\frac{d\lambda_{t}}{d\beta }$ = −8.3292 *µ_b_* + 12.438 (R^2^ = 0.7367). Because the regression coefficient had a higher value, $\frac{d\lambda_{t}}{d\beta }$ was significantly influenced by blood viscosity. Figure 7C-ii shows the linear relationship between <*λ_t_*> and *µ_b_*. Linear regression yielded a formula of <*λ_t_*> = 3.7111 *µ_b_*–3.9552 (R^2^ = 0.7047). The results confirmed that the blood viscosity contributed to <*λ_t_*> varying significantly. Finally, based on temporal variations in *I_b_* at stasis, temporal variations in AI were obtained with respect to *C_dex_*. Figure A1B (Appendix A) shows snapshots of the RBC sedimentation in the driving syringe captured at the end of the experiments (*t* = 1680 s) with respect to *C_dex_*. During the first period, the AI tended to increase at higher dextran concentrations. Over time, RBC sedimentation in the driving syringe contributed to an increase in the Hct of blood flowing in the test fluid channel. As higher Hct levels hindered RBC aggregation, the AI decreased significantly over time, particularly for higher concentrations of dextran (i.e., *C_dex_* = 15 and 20 mg/mL). Compared with Figure 7B-ii, the AI exhibited consistent trends with the time constant. The two parameters of the time constant exhibited consistent variations when compared with AI. From the experimental results, the time constant could be used to quantify the RBC aggregation with consistency. In addition, the blood viscosity (*µ_b_*) had a strong influence on the time constant (i.e., $\frac{d\lambda_{t}}{d\beta }$, and <*λ_t_*>).

Compared with previous studies [5,23,24], the present method does not assume that the time constant of blood is constant under transient blood flow. Instead of using the curve-fitting technique, it is numerically obtained by solving the governing equation. The time constant varies linearly with respect to the interface in the coflowing channel (i.e., blood flow). Next, based on the variations in the time constant over the interface, two parameters (i.e., averaged value (<λ>) and linear slope ($\frac{d\lambda }{d\beta }$)) are suggested and calculated. In the experimental results, <λ> exhibited more consistent results than $\frac{d\lambda }{d\beta }$. <*λ*> has a consistent linear relationship with the blood viscosity. Thus, instead of blood viscosity, <*λ*> could be considered an effective parameter under dynamically varying blood flow. Furthermore, it provides reciprocal trends compared with the AI. The present method can be used to detect blood with continuously varying Hct values resulting from RBC sedimentation inside a driving syringe. As a limitation of this study, the performance of the present method was validated using suspended blood rather than clinical patient blood. In future research the differences in haemorheological properties between normal and patient blood should be detected.

## 4. Conclusions

In this study, three haemorheological properties (i.e., viscosity, time constant, and RBC aggregation index) were obtained by analysing the blood flow, which was set to a square-wave profile. Two rheological properties of blood (i.e., blood viscosity and time constant) were obtained by analysing the interface in the coflowing channel. A simplified differential equation of the coflowing channel was derived using discrete fluidic circuit elements. Based on the governing equation, two properties (i.e., blood viscosity and time constant) were calculated sequentially for steady blood flow and transient blood flow. Additionally, RBC aggregation was obtained from the image intensity of the blood flow in the test fluid channel, particularly at stasis. Unlike previous studies, the time constant varied linearly with respect to the interface in the coflowing channel. Thus, two parameters (i.e., averaged value (<λ>) and linear slope ($\frac{d\lambda }{d\beta }$)) were suggested to represent trends of the time constant with respect to the interface. The experimental results indicated that <λ> provided more consistent results than $\frac{d\lambda }{d\beta }$. Blood viscosity was effective in monitoring variations in Hct rather than the two parameters of the time constant. Hardened RBCs were effectively detected in terms of either blood viscosity or the two parameters of the time constant. The present method was successfully employed to quantify variations in the Hct resulting from RBC sedimentation in a driving syringe. These three mechanical properties exhibited consistent trends with respect to the degree of RBC sedimentation. In conclusion, the present method can consistently detect variations in blood in terms of three mechanical properties of blood (i.e., blood viscosity, two parameters of time constant, and RBC aggregation).

## Data Availability

Not applicable.

## Nomenclature

$\dot{\gamma \;}$	Shear rate (s^−1^)
*λ*	Time constant (s)
*I_b_*	Image intensity of blood flow
*S_A_*, *S_B_*	Two factors estimated by temporal variations of image intensity (*I_b_*)
AI	RBC aggregation index (i.e., AI = *S_A_*/[*S_A_* + *S_B_*])
*R_t_*, *R_r_*	Fluidic resistance of test fluid and reference fluid in the coflowing channel
*W_r_*, *W_t_*	Stream width of reference fluid and test fluid
*β*	Interfacial location of test fluid (i.e., *β* = *W_t_*/[*W_t_* + *W_r_*])
*P_a_*, *P_b_*	Pressures at junction point (a, b)
*Q_t_*, *Q_r_*	Flow rate of test fluid and reference fluid
*A_c_*	Cross sectional area of microfluidic channel (i.e., *A_c_* = width [*w*] × depth [*h*])
<*U_b_*>	Averaged blood velocity
*Q_b_*	Blood flow rate (i.e., *Q_b_* = *A_c_* × <*U_b_*>)
*µ_t_*, *µ_r_*	Viscosity of test fluid and reference fluid
*C_e_*	Equivalent compliance element
*α_R_*	Correction factor of fluidic resistance (i.e., *α_R_* = *α_R_* [*β*])
*R_r_*, *R_t_*	Fluidic resistance of test fluid and reference fluid in the coflowing channel
*λ_t_*	Time constant of test fluid (i.e., *λ_t_* = $\frac{12{\;\mu }_{t}\;L}{{\mathrm{wh}}^{3}}$ × *C_e_*)
<*λ_t_*>	Average value of time constant
*dλ_t_*/*dβ*	Linear slope of time constant in relation to interfacial location.
*n*	Number of repeated experiments.
*T*	Period of blood flow with square-wave profile
*V_air_*	air cavity secured in the driving syringe
*λ_pm_*	Single value of time constant obtained with the previous method
Hct	Hematocrit of blood sample
*C_GA_*	Concentration of glutaraldehyde solution
*C_dex_*	Concentration of dextran solution
